# A study of the interaction of cationic dyes with gold nanostructures†

**DOI:** 10.1039/d1ra03459f

**Published:** 2021-05-14

**Authors:** Fengyuan Shan, Luca Panariello, Gaowei Wu, Asterios Gavriilidis, Helen H. Fielding, Ivan P. Parkin

**Affiliations:** Department of Chemistry, University College London 20 Gordon Street London WC1H 0AJ UK h.h.fielding@ucl.ac.uk i.p.parkin@ucl.ac.uk; Department of Chemical Engineering, University College London Torrington Place London WC1E 7JE UK

## Abstract

The interaction of methylene blue and crystal violet dyes with a range of gold nanoparticles (AuNPs), gold nanoclusters and gold/silver nanoclusters is reported. It is found that 20 nm citrate-capped AuNPs have strong interactions with these two dyes that result in red-shifted absorption peaks in their electronic absorption spectra. Transmission electron microscopy and dynamic light scattering measurements show that this can be attributed to these AuNPs combining into large agglomerates. Eventually, precipitation is observed. The agglomeration process is triggered when the dye reaches or exceeds a threshold concentration and then does not stop until all the AuNPs have agglomerated into large particles and precipitated. Calculations suggest that the threshold concentration corresponds to having sufficient dye molecules to form a monolayer on the surface of AuNPs. We also observe similar red-shifting in the absorption peaks of the electronic absorption spectra of 11–50 nm citrate-capped AuNPs formed by both single step and seeded growth methods. No such interactions were observed in the UV-vis spectra of the dyes with Tris-capped AuNPs, gold nanoclusters or gold/silver nanoclusters.

## Abstract 2

The interaction of methylene blue and crystal violet dyes with a range of gold nanoparticles (AuNPs), gold nanoclusters and gold/silver nanoclusters is reported.

## Graphical abstract

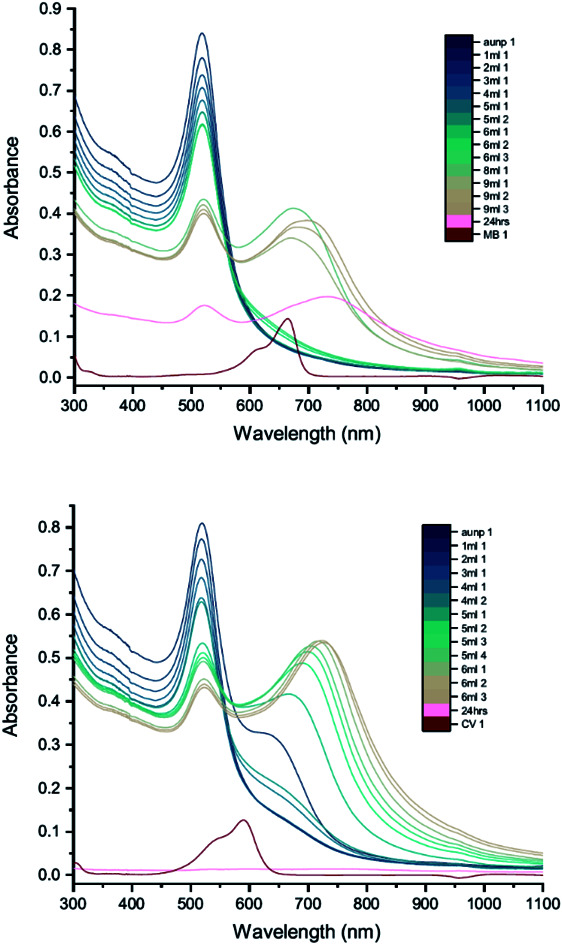

## 1 Introduction

There is a great deal of interest in improving our understanding of the interactions between metal nanostructures and adsorbate molecules. One particular example is the interaction between gold nanoparticles (AuNPs) and photosensitiser dyes. When a photosensitiser dye, like crystal violet (CV) or methylene blue (MB), is irradiated with light, it generates lethal photosensitisation by creating reactive oxygen species (ROS) which kill bacteria.^1^ Photosensitisation has been found to be enhanced when these dyes are combined with a range of substrates,^2,3^ including AuNPs.^4^ The interaction between the dye and the substrate enhances light harvesting by the dye so it produces greater concentrations of ROS and therefore has an enhanced anti-microbial effect. The interaction between CV and gold nanoclusters (AuNCs) has a similar effect,^5^ although AuNCs seem to alter the pathway of the dye sensitization process and specifically generate hydrogen peroxide^6^ rather than the mixture of ROS that is seen for dye–AuNP systems. In order to understand how combining dyes, such as CV or MB, with AuNPs and AuNCs enhances the generation of ROS, it is important to improve our understanding of the dye–gold interaction. Here we build on earlier work,^7,8^ investigating how interactions between dyes and AuNCs and AuNPs of various size and surface chemistry enhance UV-visible absorption, investigating how the dye–gold interaction is affected by changes in these parameters. To this aim, we synthesized AuNCs and NPs according to various protocols,^9–11^ enabling control of particle features such as size and surface chemistry.

Studies of interactions between dyes and nanostructures have been undertaken since 1999. Khazraji *et al.* reported an interaction between dyes and nanostructured TiO_2_ films that resulted in agglomeration and an enhanced photosensitisation efficiency of the anionic cyanine dye Merocyanine 540.^12^ In the case of cationic dyes, Narband *et al.* reported that the mixing of citrate-capped AuNPs with the thiazine family of cationic dyes enhanced the absorption coefficient of the dye.^7^ There are also numerous reports of morphological changes when AuNPs are mixed with cationic dyes, with the AuNPs undergoing agglomeration to form large clusters.^7,8,13^

Previous work has focussed solely on citrate capped AuNPs in an acidic environment.^7,8^ Here, we extend this work to investigate the effect of changing the pH of the AuNP solution. Moreover, we show that the process is triggered when the dye reaches or exceeds a threshold concentration and then does not stop until all the AuNPs have agglomerated into large particles and precipitated. This observation has not been reported before. To study the electronic interactions between Au nanostructures and dyes, we use UV-vis absorption spectra to monitor if there are any changes in wavelength. To investigate the changes in morphology that accompany these interactions, we use transmission electron microscopy (TEM) and dynamic light scattering (DLS) measurements of particle size. We employ nanostructures with narrow size distributions ranging from very well-defined NCs (Au_25_ or Au_16_Ag_9_) to larger AuNPs (11–50 nm diameter, 10 000–200 000 Au atoms). We show that all the AuNPs synthesized using citrate groups as capping agents have strong interactions with CV and MB that enhance the UV-vis absorption of the dyes, but that AuNPs synthesized with Tris-base capping agents and AuNCs do not interact with CV and MB to enhance the absorption of the dyes.

## 2 Experimental methods

### 2.1 Nanomaterial synthesis

A detailed description of the experimental protocols employed to synthesise the NCs and NPs used in this work (Table 1) can be found in Section S1.† Briefly, Au and Au/Ag NCs were produced using the procedure reported by Hwang *et al.*^11^ with CO as a reductant and cysteine as the co-ordinating ligand. This procedure leads to the production of Au_25_ NCs (*i.e.*, having 25 gold atoms).^11^ 11 nm citrate-capped AuNPs were prepared with a “passivated method” in a flow reactor using a basic Au(OH)_4_^−^ precursor reduced with citric acid, following the procedure reported by Panariello *et al.*^10^ These particles were also used as seeds to produce larger AuNPs *via* seeded growth protocols. 50 nm Tris-capped AuNPs were produced using a flow method developed by Panariello *et al.*;^9^ the 11 nm citrate-capped nanoparticles were grown with the addition of Au(iii) precursor reduced by H_2_O_2_ with tris(hydroxymethyl)aminomethane (Tris-base) as the capping agent. 25 nm citrate-capped AuNPs were produced in batch with a seeded-growth approach, starting from the 11 nm citrate-capped AuNPs. These were grown using an extension of the passivated method from Panariello *et al.*,^10^ with Au(OH)_4_^−^ as the precursor and citric acid as the reducing agent. 20 and 50 nm citrate-capped AuNPs were produced using an iterative seeded growth approach with hydroxylamine as the reducing agent, following the method reported by Brown *et al.*^15^ and using the 11 nm citrate-capped AuNPs as seeds.

**Table 1:** Gold nanoparticles synthesised for this work

Sample name	Reducing agent	Capping agent	Size
20 nm citrate-capped AuNPs^14,15^	Hydroxylamine	Citrate	20 nm
50 nm citrate-capped AuNPs^14,15^	Hydroxylamine	Citrate	50 nm
50 nm Tris-capped AuNPs^9^	H_2_O_2_	Tris-base^a^	50 nm
25 nm citrate-capped AuNPs^10^	Citric acid	Citrate	25 nm
11 nm citrate-capped AuNPs^10^	Citric acid	Citrate	11 nm
Cysteine-protected AuNCs^11^	CO	Cysteine	<2 nm
Cysteine-protected Au/AgNCs^11^	CO	Cysteine	<2 nm

a Tris(hydroxymethyl)aminomethane.

### 2.2 UV-vis peak development measurements

The UV-vis peak development measurements reported in this work are based on published procedures.^7,8^ For AuNPs, the solutions were diluted until the absorbance of the LSPR band around 530 nm was in the range *A* = 0.5–1.0. Afterwards, a 16 mL aliquot was transferred into a centrifuge tube. 1 mL of dye solution of known concentration was added, and the mixture stirred. Immediately, some solution was transferred into a cuvette and a UV-vis spectrum recorded over the range 300 nm to 1100 nm (1 nm resolution) using a PerkinElmer Lambda 365 spectrometer.

The solution in the cuvette was then poured back into the centrifuge tube and an additional 1 mL of dye solution added. This whole procedure was repeated 15 times or until the peak development in the UV-vis spectrum was complete. This process assumed that the dye was the only factor in the interaction and that the process stopped when it reached a stable state. We adapted the procedure to record each spectrum twice instead of once if we found that after an interaction was triggered, the reaction was complete before further dye could be added.

### 2.3 Particle size measurements

TEM measurements were performed using a JEOL 2010 microscope. The operating power was 200 kV and image collection, and processing were performed on a CCD with Gatan Digital Micrograph software. Particle size analysis was also undertaken. DLS measurements were performed with a DelsaMax-Pro (Beckman Coulter) at 22 °C. The acquisition times were set to be 5 × 5 s; each sample was tested six times. DLS measurements provide evidence of hydrodynamic diameter changes of particles.

### 2.4 Calculation of dye coverage on AuNPs

Calculation of the dye coverage was achieved by assuming that the AuNPs were spherical and packed analogously to bulk gold. The surface areas of MB and CV dye molecules were approximated using three models: (a) van der Waals surface area (VDWSA),^16^ (b) solvent accessible surface area (SASA)^17^ and (c) space filling projection.^17^ The volumes of dye required for forming one monolayer on each gold nanoparticle in solution were then calculated, noting that surface areas determined using VDWSA and SASA models need to be halved since it is not possible for a dye molecules to project more than half of its surface area onto the AuNP.

## 3 Results and discussion

The initial focus of this work is on 20 nm citrate-capped AuNPs, for which in addition to UV-vis spectra, we also present TEM and DLS measurements and investigate different pH environments. We then investigate the role of the capping and reducing agents employed in the synthesis of AuNPs with narrow size distributions across the range 11–50 nm, for which we present UV-vis peak development measurements. We also report UV-vis peak development measurements for Au and Au/Ag NCs.

### 3.1 UV-vis measurements

**Fig. 1:**
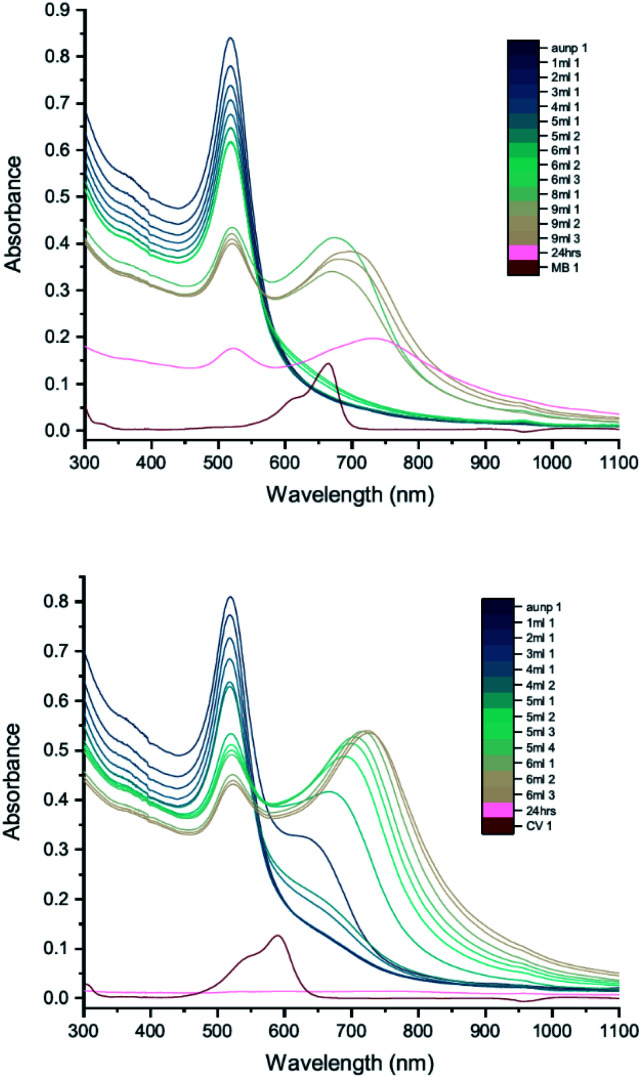
UV-vis absorption of 2 mM MB (top) and 1.5 mM CV (bottom) in solutions of 20 nm citrate-capped AuNPs. The red curve is the absorption spectrum of the dye. The other curves represent spectra recorded following the addition of 1–9 mL dye solution (the numbers at the ends of the labels in the key state the measurement number).

The results of UV-vis peak development measurements for 20 nm citrate-capped AuNPs, synthesised using hydroxylamine as the reducing agent, with 2 mM MB and 1.5 mM CV, are presented in Fig. 1 (and Fig. S1† in which the dye contribution has been subtracted). For the measurements with MB, those with the first 4 mL were similar to the original AuNP solution. After 5 mL was added, some enhancement in the 600–750 nm range was observed and after 6 mL dye was added, a new peak centred around 720 nm appeared. Following the addition of more dye, this peak shifted to longer wavelengths. This new peak became as intense as that of the localized surface plasmonic resonance (LSPR)^18–21^ peak, at 520 nm, after 9 mL MB had been added. Similar observations were made for measurements with CV, although the new peak appeared much more quickly, after the addition of only 2 mL. This new peak became more intense than the LSPR peak after 6 mL CV had been added and red-shifted to around 730 nm. After 24 h, strong precipitation was observed in both MB and CV measurements and the extinction decreased dramatically. These observations are similar to those reported previously,^7,8^ and can be attributed to agglomeration of the NPs; however, it is worth noting that the new peaks observed in these new measurements, with 20 nm citrate-capped AuNPs, appear at longer wavelengths than the earlier measurements with 11 nm AuNPs^7,8^ (600–700 nm).

We can estimate concentrations of MB and CV molecules required to form monolayers on the surface of the AuNPs. The number density of AuNPs was determined using:1
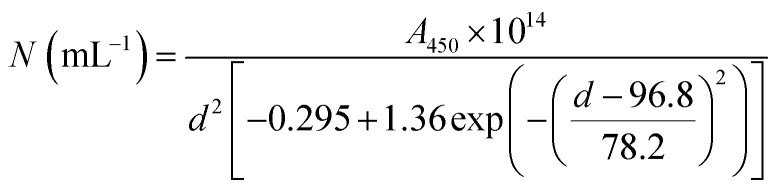
Eqn (1), derived by Haiss *et al.*,^22^ has been shown to work well for AuNPs < 100 nm. *A*_450_ is the absorbance at *λ* = 450 nm and *d* is the particle diameter in nm. From Fig. 1, we deduce that *A*_450_ = 0.48 and 0.49 for MB and CV, respectively, giving *N* = 5.37 × 10^11^ mL^−1^ and 5.48 × 10^11^ mL^−1^ for MB and CV, respectively. The surface area of MB is estimated to be 3.63 nm^2^ (VDWSA), 6.01 nm^2^ (SASA) or 0.93 nm^2^ (SFP), and that of CV is estimated to be 7.46 nm^2^ (VDWSA), 10.56 nm^2^ (SASA) or 1.72 nm^2^ (SFP). From these values, we can calculate the concentration of dye required for monolayer coverage of the AuNPs and compare these with the threshold concentrations determined from the UV-vis spectra (Table 2 and Section S2†).

**Table 2:** Comparison between the calculated dye concentration required for monolayer coverage of the AuNP surface compared with the threshold concentration determined from UV-vis absorption spectra (Fig. 1 and 6 for 20 nm and 50 nm citrate-capped AuNPs, respectively, with curve labels in parentheses)

	Model	20 nm citrate-capped AuNP		50 nm citrate-capped AuNP	
		MB	CV	MB	CV
Concentration of dye/μM	VDWSA	0.62	0.31	0.14	0.05
	SASA	0.37	0.22	0.08	0.04
	SFP	1.21	0.67	0.27	0.12
Experimental threshold conc./μM		0.55	0.36	0.22	0.09
Corresponding solution vol./mL		6.0	5.0	2.0	1.0

From Table 2, we see that for both MB and CV, the triggering threshold is located around the point where the dye molecules could form a monolayer coverage on the surface of the AuNPs. We also see similar results using an alternative procedure^23^ (Section S3†).

### 3.2 TEM

**Fig. 2:**
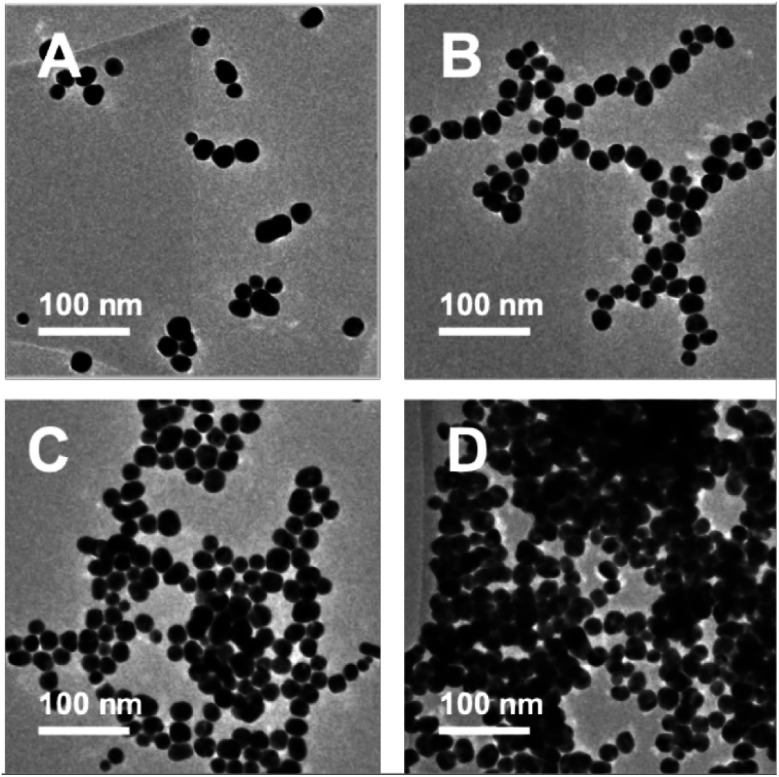
TEM images of the AuNPs obtained during the dilution process between 2 mM MB and 20 nm citrate-capped AuNPs after the addition of 0 mL (A), 5 mL (B), 10 mL (C) and 15 mL (D) dye.

**Fig. 3:**
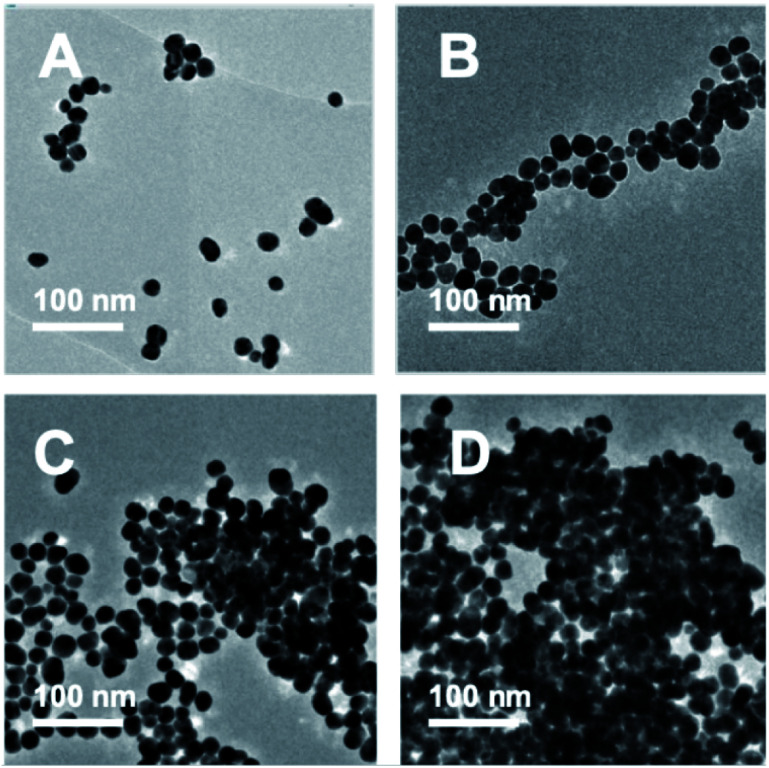
TEM images of the AuNPs obtained during the dilution process between 1.5 mM CV and 20 nm citrate-capped AuNPs after the addition of 0 mL (A), 5 mL (B), 10 mL (C) and 15 mL (D) dye.

TEM images were recorded to see if there were any obvious differences in morphology (Fig. 2, 3 and Table S2†). TEM images of the 20 nm citrate-capped AuNPs present in the solution both before and after the addition of 0–15 mL MB and CV show the process of agglomeration. Representative images are shown, but similar patterns of AuNPs are observed across the whole TEM grid. In Fig. 2D and 3D, it is clear that large clusters are formed but that the majority of particles are still quasi-spherical, and the morphology is similar to that observed in Fig. 2C and 3C. TEM measurements are not always representative of what is happening in solution as clustering can occur on drying samples onto the TEM grids. However, it is worth noting that we only saw agglomeration on the TEM grids in those samples where the UV-vis and DLS measurements indicated agglomeration.

### 3.3 DLS

**Fig. 4:**
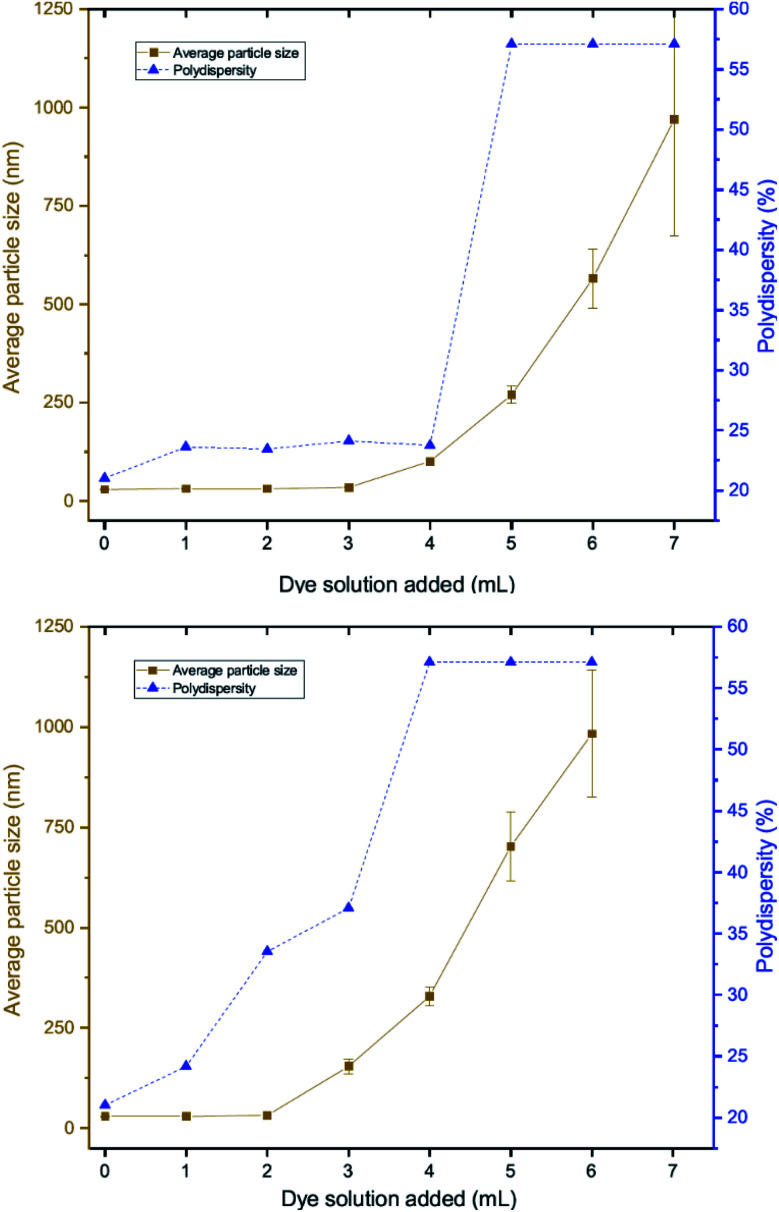
DLS measurements of solutions obtained during the dilution process between 2 mM MB (top) or 1.5 mM CV (bottom) and 20 nm citrate-capped AuNPs showing polydispersity (blue) and hydrodynamic particle sizes (brown).

DLS measurements were also performed as a function of volume of dye solution added to the 20 nm citrate-capped AuNPs to investigate whether this type of agglomeration was caused by surface drying (Fig. 4). Both the hydrodynamic sizes of the particles and the polydispersity increased dramatically with addition of dye. For MB, the average hydrodynamic diameter was measured to be 30 nm before addition of dye, but it increased to 101 nm after the addition of just 4 mL CV solution. After the addition of 6 mL, the average hydrodynamic size increased to 565 nm, providing evidence of strong agglomeration of the particles in solution. Since the hydrodynamic size measured by DLS is obtained assuming all particles are spherical, random agglomeration explains the large error bar for the 5 mL to 7 mL measurements and the off-scale polydispersity. There is a correlation between the DLS measurements and the UV-vis spectra. The red-shifting interaction peak also shows up after 4 mL solution has been added, supporting the suggestion that the interaction peak is due to the agglomeration. Similar experiments were carried out using CV with similar results. Notably, the TEM images show that the citrate-capped NPs do not undergo Ostwald ripening but stay the same size during the agglomeration process. However, from both the TEM grids and the DLS measurements, it is clear that the AuNPs cluster together to a greater extent with increasing dye content. This clustering seems to correlate with the changing the UV-vis profile (Fig. 1).

**Fig. 5:**
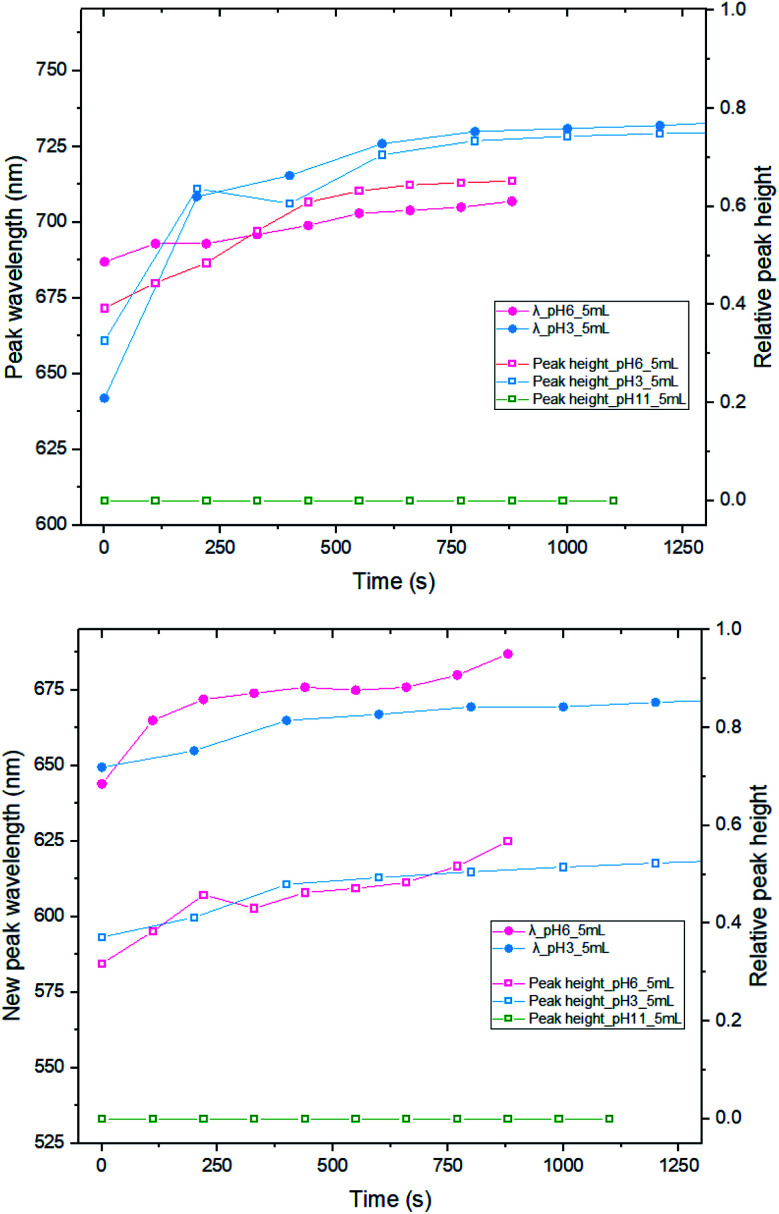
New peak position and relative peak height measurements between 5 mL of 2 mM MB (top) or 1.5 mM CV (bottom) and 16 mL of 20 nm citrate-capped AuNPs under different pH conditions. The relative peak height is the ratio of new peak absorbance and absorbance of LSPR peak in the original AuNPs solution.

### 3.4 pH

We have also investigated the effect of changing the pH, from pH 3 (original) to pH 6 and 11, by adding 200 μL and 600 μL of 0.1 M NaOH, respectively (Fig. 5). Immediately after mixing 5 mL of MB/CV with 20 nm AuNPs solution, spectra were recorded after several time intervals. The location of the new peak and the absorbance wavelength were recorded. By comparing the results of both the new peak wavelength and relative intensity (the ratio between the new peak absorbance and original LSPR absorbance), it is apparent that there is no significant difference on development trend between running the experiment with pH 3 or 6. However, when the pH was 11, we did not observe any new peaks in the UV-vis spectra and the pattern is indicative of a simple addition of dye and AuNPs, suggesting that there is no interaction between AuNPs and dyes at pH 11. We speculate that the dye species may change its charge to become negative and that is why it does not interact with the negatively charged nanoparticles. Alternatively, it could be the higher ionic strength associated with the higher pH enhances the shielding between the AuNPs and dyes.

### 3.5 AuNP size

**Fig. 6:**
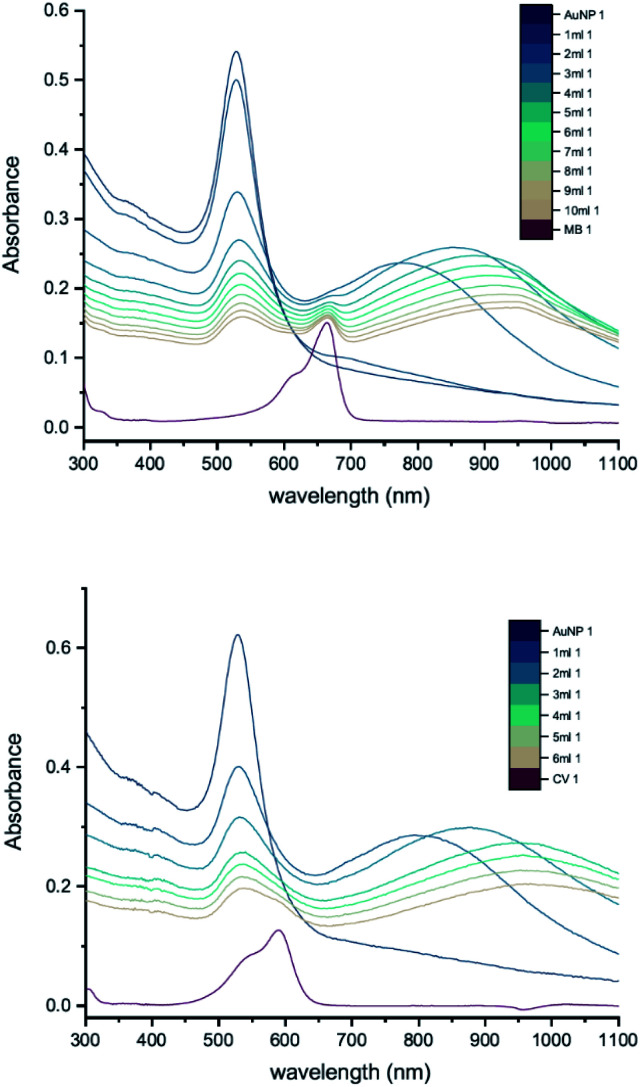
UV-vis absorption of 2 mM MB (top) and 1.5 mM CV (bottom) in solutions of 50 nm citrate-capped AuNPs. The red curve is the absorption spectrum of the dye. The other curves represent spectra recorded following the addition of 1–10 mL dye solution (the numbers at the ends of the labels in the key state the measurement number).

To investigate the influence of AuNP size, we measured the UV-vis peak development for 50 nm citrate-capped AuNPs, also synthesised using hydroxylamine as the reducing agent, with 2 mM MB and 1.5 mM CV. Fig. 6 shows the results of these experiments. As with the 20 nm citrate-capped AuNPs, the 50 nm AuNPs exhibited a strong interaction. For the measurements with 50 nm citrate-capped AuNPs and MB, the spectrum with the initial 1 mL dye solution was similar to the original AuNP solution, despite some enhancement in the 650–750 nm range. However, after the second 1 mL dye solution was added, the LSPR band at 530 nm decreased dramatically, much lower than for simple dilution, and a new peak appeared in the longer wavelength region around 750 nm. After adding more dye solution, the LSPR peak continued to drop slowly and the new peak kept red-shifting from 750 nm to 930 nm. The extinction of this new peak was highest when 3 mL of dye solution was added, after which it dropped slowly, similar to the LSPR peak. For the measurements with 50 nm citrate-capped AuNPs and CV, a very significant interaction occurred immediately after the addition of 1 mL dye solution. The extinction of the LSPR peak decreased by almost one third, from 0.62 to 0.40. A new peak also appeared, with a maximum around 790 nm. After this, the trend was similar to MB. The extinction of the new peak was highest following the addition of 2 mL of CV after which it reduced slowly, similar to the LSPR band, and it continued to red-shift from 800 nm to greater than 930 nm. The longest wavelengths of the new peaks in the measurements made with 50 nm AuNPs are longer than those observed in the measurements made with 20 nm AuNPs (700–750 nm). As with the 20 nm AuNPs, the triggering threshold is still located around the point where the dye molecules are calculated to form a monolayer coverage on the surface of the 50 nm citrate-capped AuNPs (Table 2).

**Fig. 7:**
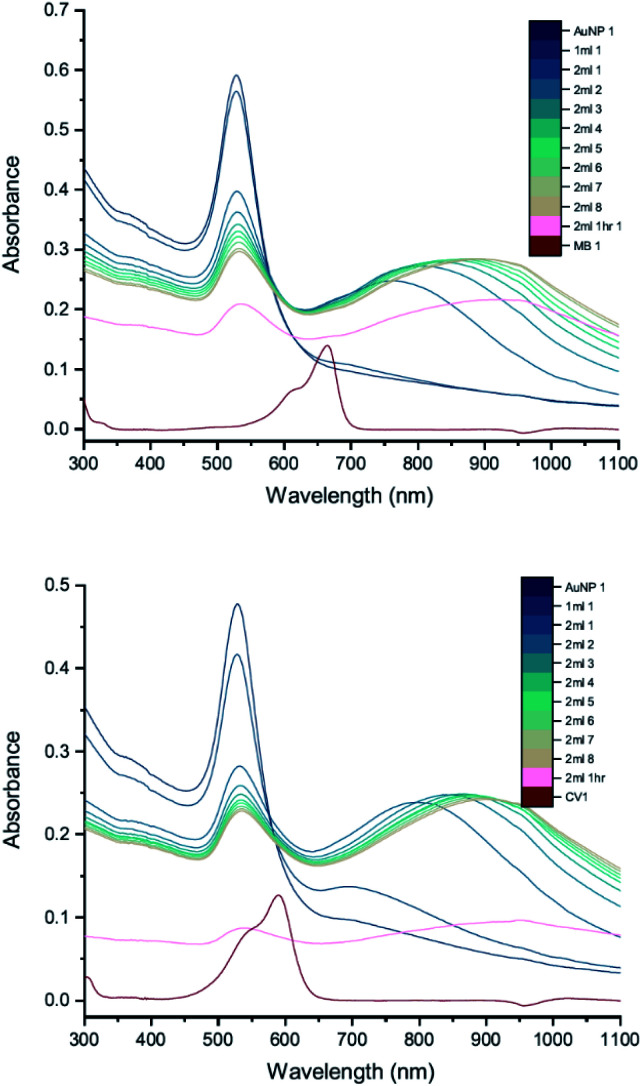
UV-vis absorption of 2 mM MB (top) and 1.5 mM CV (bottom) in solutions of 50 nm citrate-capped AuNPs tracking the peak development after the addition of 2 mL dye. The red curve is the absorption spectrum of the dye. The other curves represent spectra recorded following the addition of 1 or 2 mL dye solution (the numbers at the ends of the labels in the key state the measurement number).

Whilst the UV-vis spectra were recorded, the colour of the solution in the centrifuge tube was observed to change from wine-red to purple, hinting that the interaction process was ongoing and had not reached a stable state. This suggests that increasing the amount of dye may not be the only cause of the interaction. To investigate this, peak development tests were carried out (Section 2.2). As soon as the new peak appeared, no more dye was added but UV-vis spectra of the solution in the cuvette were recorded many times, until the peak development was shown fully. Fig. 7 shows the results of such a peak development test for 50 nm citrate-capped AuNPs. Both dyes show strong interactions after addition of 2 mL of dye solution. The trend of the new peak is similar to the ones using the same dye solution in Fig. 6. The maximum of the new peak red-shifted slowly, from 750 nm to 930 nm for MB and from 780 nm to 930 nm for CV. The peak height remained constant for 8 UV-vis spectra, which is equivalent to 30 minutes. The peak height of the LSPR band dropped more slowly than was observed in Fig. 6; however, if the dilution effect of the extra dye solution is taken into account, the peak development is very similar to those observed in Fig. 6. After 1 hour, UV-vis spectra were recorded again and the absorbance was found to have dropped dramatically and precipitation had occurred, indicating strong agglomeration.

### 3.6 Capping agent

**Fig. 8:**
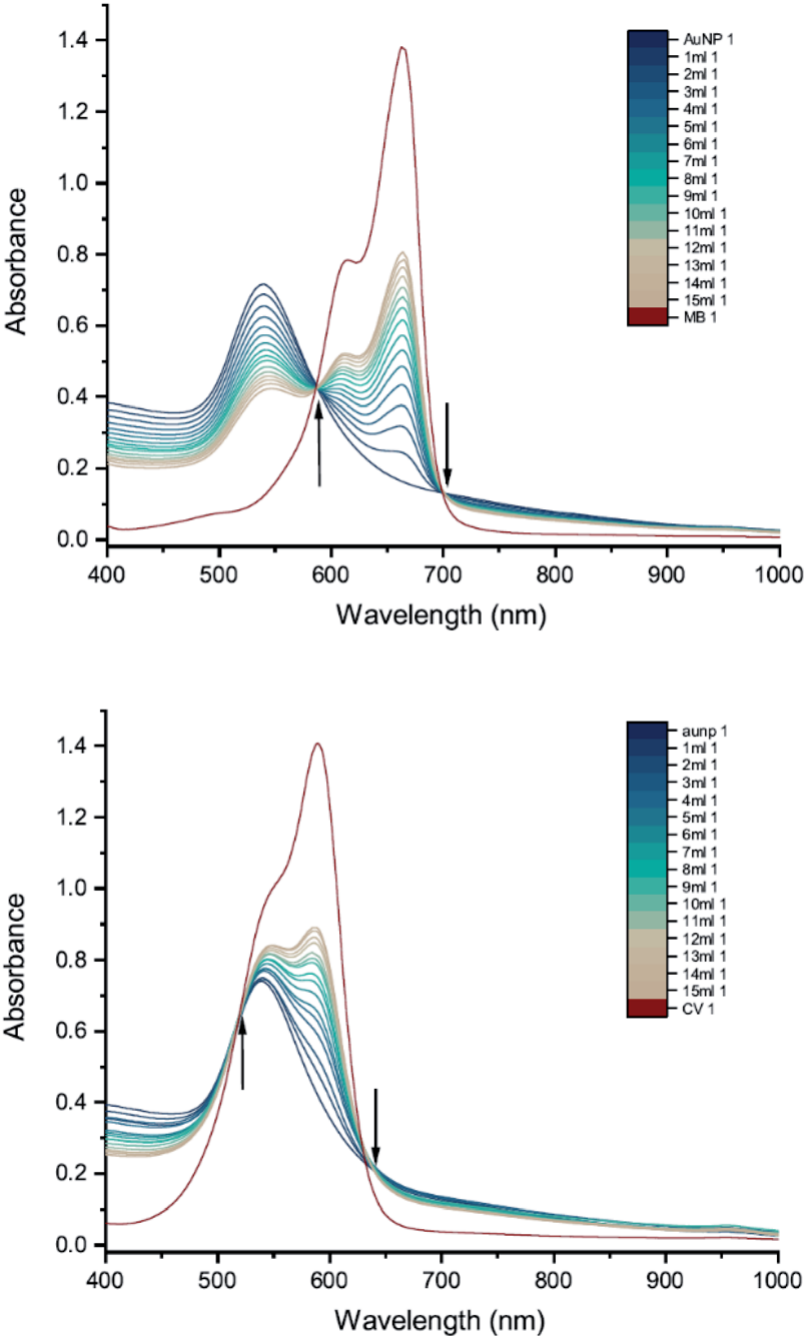
UV-vis absorption spectra of 20 mM MB (top) and 15 mM CV (bottom) in solutions of 50 nm Tris-capped AuNPs. The red curve is the absorption spectrum of the dye. The other curves represent spectra recorded following the addition of 1–15 mL dye solution (the numbers at the ends of the labels in the key state the measurement number). Black arrows point towards the isosbestic points.

To investigate how the capping agent used in the AuNP synthesis affects the AuNP–dye interactions, we carried out UV-vis peak development measurements with 50 nm Tris-capped AuNPs and 20 mM MB or 15 mM CV (Fig. 8). It should be noted that the synthesis of these AuNPs also used a different reducing agent, H_2_O_2_. Although H_2_O_2_ is generally considered as an oxidizing agent, it can reduce Au(iii) to Au(0) over a broad range of pH values,^24^ favouring particle growth over homogeneous nucleation when used to reduce Au(iii) in the presence of Au seeds.^9,25,26^ The LSPR band is observed around 535 nm. Compared to 50 nm citrate-capped AuNPs (Fig. 6), the spectra do not have new peaks appearing. Moreover, as the dye was added, patterns of simple dilution were observed: the LSPR band decreased with addition of dye solution whilst the absorbance peak associated with the dye increased and isosbestic points were observed, which indicates that there was only a pure addition effect of dye and AuNPs. It is worth mentioning that the pH of the 50 nm Tris-capped AuNPs solution is around 4, which is in the range where we observed agglomeration for the 20 nm citrate-capped AuNPs.

The difference between the Tris-capped and citrate-capped AuNPs can most likely be attributed to a different charge balance between the AuNPs and dyes. The interplay between the coulombic attraction and steric repulsion originating from the surface coating layers is expected to impact agglomeration kinetics.^27^ For Tris-capped AuNPs, the chelating Tris-base ligand is expected to be more tightly attracted to the AuNPs, as it is a tridentate ligand.^9^ On the other hand, the citrate ligand is not as strong and the binding mode of citrate in the stabilization of AuNPs highly depends on the citrate : Au ratio, which is much more readily affected by the environment.^28^ Thus, although N–Au interactions are relatively weak, having three per molecule is likely to offer better adherence and protection against an incoming dye than the citrate-capped NPs. Moreover, the absolute value of zeta potential for Tris-capped AuNPs is higher than that for citrate-capped AuNPs^29^ and thus likely to result in a reduced interaction with cationic dyes.

### 3.7 Reducing agent

**Fig. 9:**
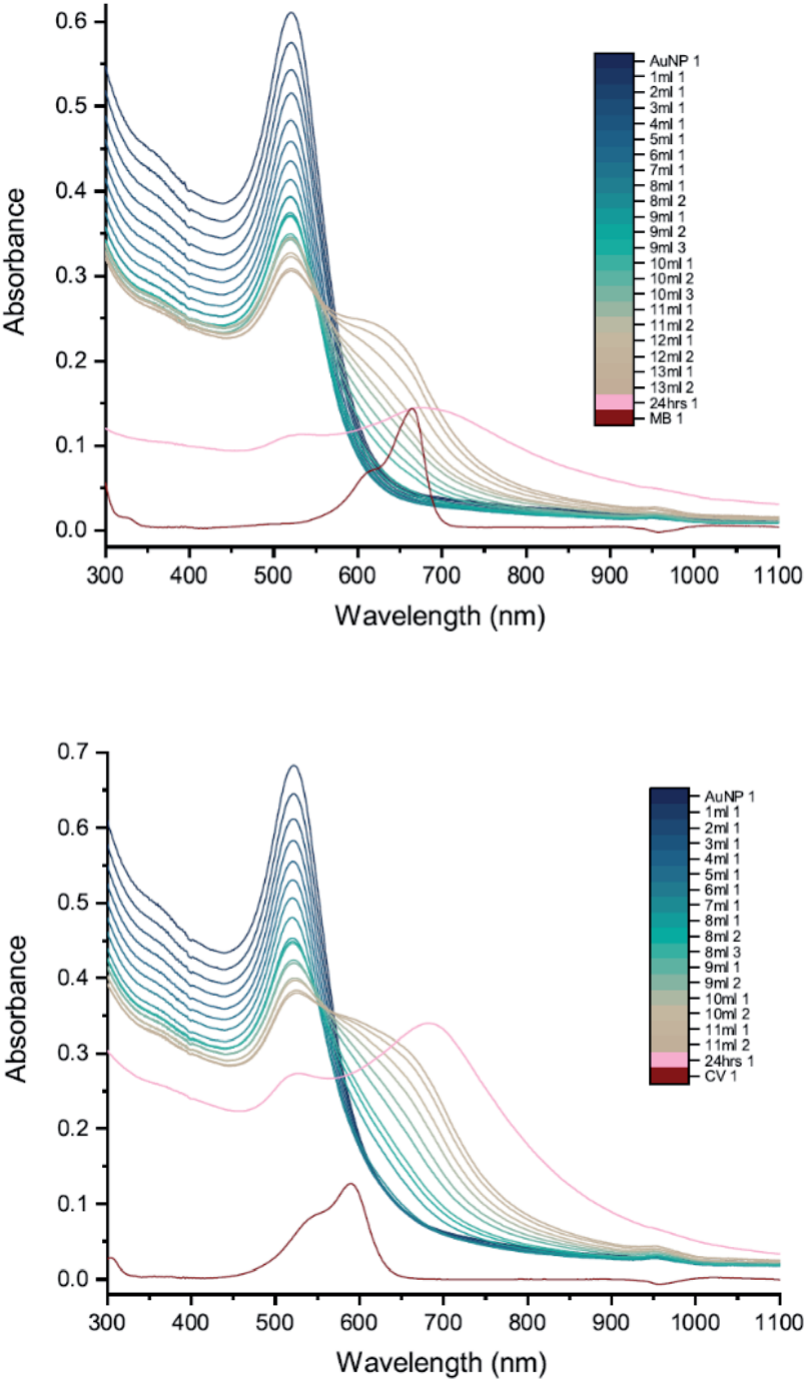
UV-vis absorption spectra of 2 mM MB (top) and 1.5 mM CV (bottom) in solutions of 11 nm citrate-capped AuNPs. The red curve is the absorption spectrum of the dye. The other curves represent spectra recorded following the addition of 1–13 mL dye solution (the numbers at the ends of the labels in the key state the measurement number).

In order to compare more directly with the earlier measurements of AuNP–dye interactions,^7,8^ measurements were carried out using 11 nm citrate-capped AuNPs, synthesised using citric acid as a reducing agent (Fig. 9). This type of AuNP is very similar to those used in the earlier work, both in terms of size and structure; however, the dye concentrations are lower in our measurements (2 mM of MB and 1.5 mM of CV). The UV-vis peak development measurements with 11 nm AuNPs showed that changes were less pronounced than with the solutions of larger AuNPs. The wavelength of the interaction peak was also shorter. This is most likely because higher concentrations of smaller AuNPs are required to achieve the same extinction as solutions of larger AuNPs. The total surface area is then also larger and thus the volume of the citrate group is larger, requiring more dye to overcome the energy barrier for agglomeration to happen. Nonetheless, after adding enough dye solution, the AuNPs and dye are no longer in a stable state and the agglomeration process is triggered. It is clear from the spectra that once the interaction is triggered, the extinction can be enhanced either by adding more dye solution or waiting for longer. After 24 hours, the spectra developed some unique features. The LSPR peak blue-shifted to 450 nm and the new peak red-shifted to 760 nm with very high extinction. Although precipitation was observed for 11 nm, 20 nm and 50 nm citrate-capped AuNPs, the amount of precipitation for a given volume increases with AuNP size.

**Fig. 10:**
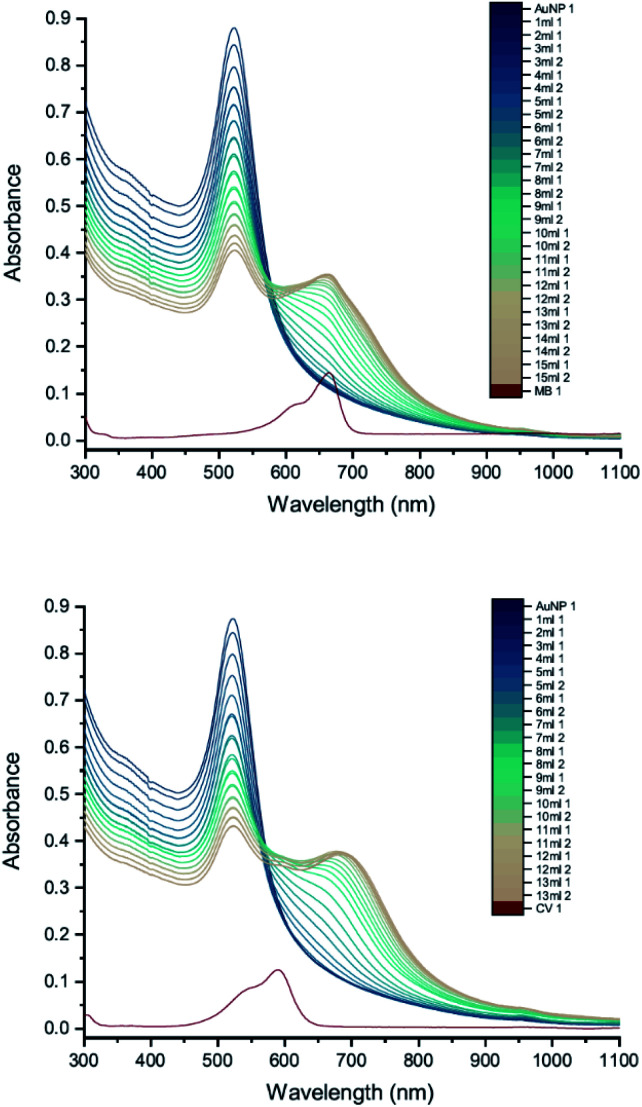
UV-vis absorption of 2 mM MB (top) and 1.5 mM CV (bottom) in solutions of 25 nm citrate-capped AuNPs. The red curve is the absorption spectrum of the dye. The other curves represent spectra recorded following the addition of 1–15 mL dye solution (the numbers at the ends of the labels in the key state the measurement number).

To check the influence of reducing agent used in the synthesis of AuNPs, measurements were carried out with 25 nm citrate-capped AuNPs, synthesised using citric acid as the reducing agent, and compared with the 20 nm citrate-capped AuNPs, synthesised using hydroxylamine as the reducing agent. UV-vis peak development measurements for these 25 nm citrate-capped AuNPs and 2 mM of MB and 1.5 mM of CV are presented in Fig. 10. For the 25 nm citrate-capped AuNPs, the LSPR peak decreases more slowly and the new peak appears and red-shifts more slowly than for the 20 nm citrate-capped AuNPs. However, the LSPR peak decreases more slowly and the new peak develops faster than for 11 nm citrate-capped AuNPs, synthesised with citric acid as the reducing agent. For the 25 nm citrate-capped AuNPs, the wavelength of the new peak was around 650 nm for MB and 680 nm for CV, which is in between the wavelengths for the 11 nm and 20 nm AuNPs. The precipitation also appeared more slowly than for the 20 nm AuNPs; nonetheless, most of the AuNPs precipitated out within 48 hours. These observations suggest that the reducing agent employed in the synthesis does influence the agglomeration process.

### 3.8 Nanoclusters

**Fig. 11:**
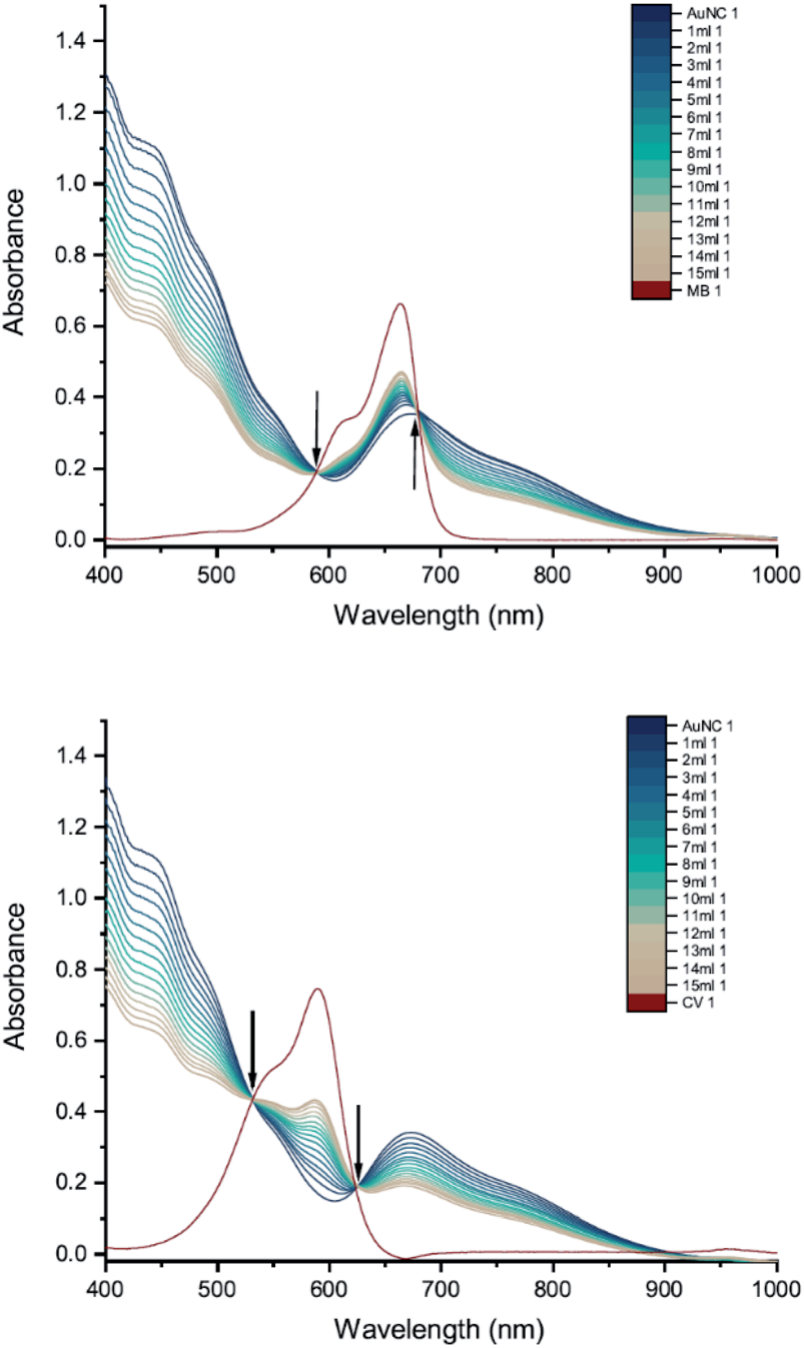
UV-vis absorption spectra of 8 mM MB (top) and 10 mM CV (bottom) in solutions of cysteine-protected AuNCs. The red curve is the absorption spectrum of the dye. The other curves represent spectra recorded following the addition of 1–15 mL dye solution (the numbers at the ends of the labels in the key state the measurement number). Black arrows point towards the isosbestic points.

Finally, we carried out UV-vis peak development measurements with gold nanoclusters because they have been shown to enhance the antimicrobial effect of CV.^11^ Solutions of cysteine-protected AuNCs were diluted to 1 mM. For the UV-vis peak development measurements, the concentrations of MB and CV were 8 mM and 10 mM, respectively. The UV-vis measurements show patterns of simple dilution (Fig. 11): the peaks associated with the AuNCs, corresponding to intraband (sp → sp) and mixed interband (d → sp) transitions,^30^ decrease with addition of dye solution whilst the absorbance peak associated with the dye increases and isosbestic points are observed.

**Fig. 12:**
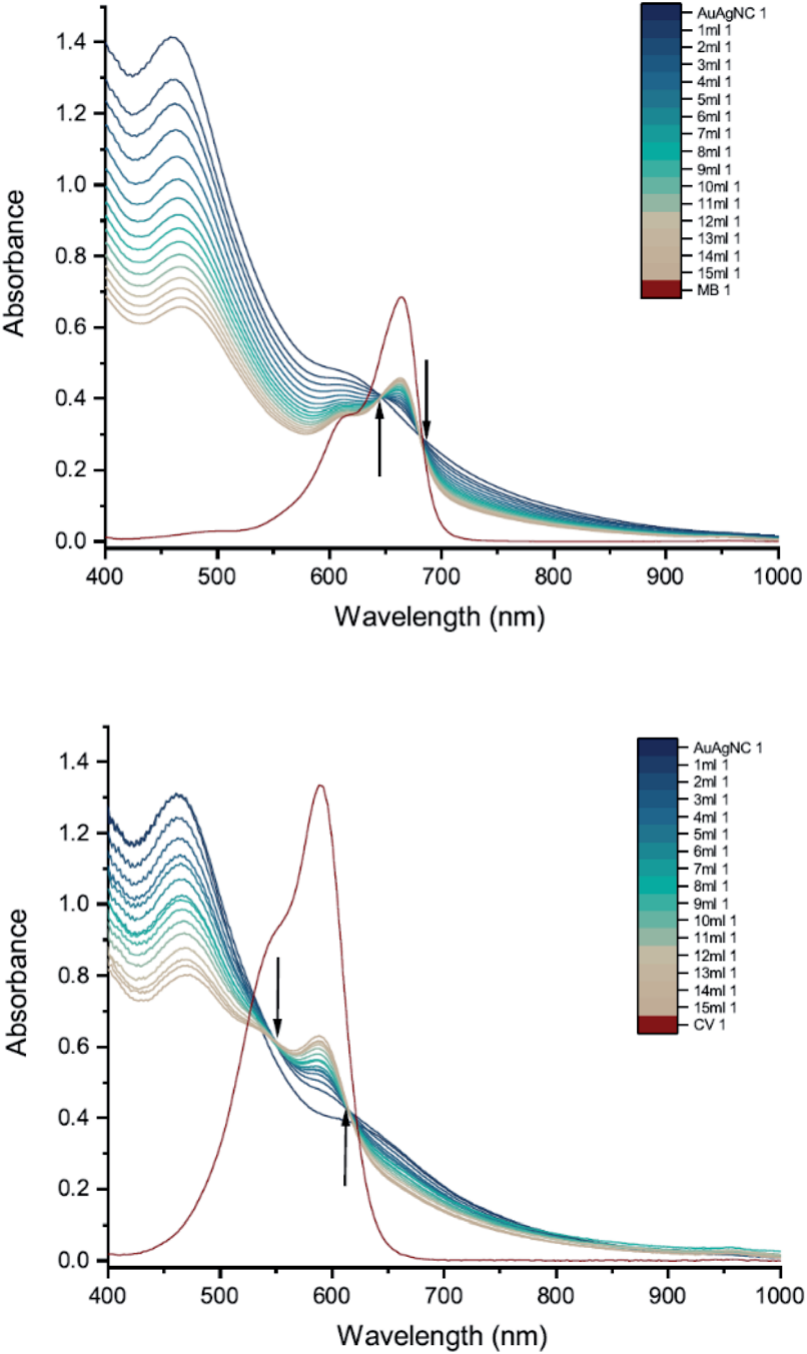
UV-vis absorption spectra of 8 mM MB (top) and 15 mM CV (bottom) in solutions of cysteine-protected Au/Ag NCs. The red curve is the absorption spectrum of the dye. The other curves represent spectra recorded following the addition of 1–15 mL dye solution (the numbers at the ends of the labels in the key state the measurement number). Black arrows point towards the isosbestic points.

Cysteine-protected Au/Ag NCs (Au : Ag ratio 16 : 9) diluted to 1 mM were also studied (Fig. 12). The dye concentrations used in the UV-vis peak development measurements were 8 mM for MB and 15 mM for CV. Similar to the AuNCs, the UV-vis measurements show patterns of simple dilution.

Thus, the interaction between AuNCs and CV that results in an enhanced anti-microbial effect does not manifest itself in the UV-vis absorption spectrum in the same way as the interaction between AuNPs and CV. We suspect this can be attributed to the sulphur-containing cysteine group being more tightly bound to the cluster, since gold forms uniquely strong interactions with sulphur compared to any other element, which restricts the close approach or coordination of dye molecules to the Au or Au/Ag core.

## 4 Summary

We have shown that the interaction previously observed between cationic dyes and 11 nm citrate-capped AuNPs^11,12^ is also found in other citrate-capped AuNPs. The interactions between the dyes and AuNPs were observed as new peaks in the UV-vis absorption spectra that appear when there is enough dye to form a monolayer on the surface of AuNPs. These peaks appeared at longer wavelengths for larger AuNPs synthesised with the same reducing agent and the absorption of these peaks increased with AuNP size. Agglomeration of 20 nm AuNPs was observed and correlated with the development of the new peak in the UV-vis spectrum. For Tris-capped NPs and cysteine-protected NCs, we did not observe any new features in the UV-vis spectra. For the AuNCs, this is interesting since earlier work has shown that cysteine-protected AuNCs and photosensitiser dyes produce potent low-light level photosensitisation, similar to the citrate-capped AuNPs. One of the goals of our future work will be to improve our molecular-level understanding of how the interaction between the dyes and gold nanostructures impacts the electronic structure and of the mechanism of photosensitisation enhancement, which will allow us to design more efficient light-activated anti-microbial coatings from first principles.

## Conflicts of interest

There are no conflicts to declare.

## Supplementary Material

RA-011-D1RA03459F-s001
