# Supplementary material for: A study of the interaction of cationic dyes with gold nanostructures
Source: RSC Adv. 2021 May 14;11(29):17694–703. doi: 10.1039/d1ra03459f (PMC9033244; doi:10.1039/d1ra03459f)
Supplement: RA-011-D1RA03459F-s001 [file RA-011-D1RA03459F-s001.pdf]

Electronic Supplementary Material (ESI) for RSC Advances.
This journal is © The Royal Society of Chemistry 2021

A Study of the interaction of cationic dyes with gold nanostructures

Fengyuan Shan,[a] Luca Panariello,[b] Gaowei Wu,[b] Asterios Gavriilidis,[b] Helen H. Fielding*[a] and Ivan P. Parkin*[a]

[a]*Department of Chemistry, University College London, 20 Gordon Street, London, WC1H 0AJ, UK*
[b]*Department of Chemical Engineering, University College London, Torrington Place, London WC1E 7JE, U. K.*

E-mail: h.h.fielding@ucl.ac.uk; i.p.parkin@ucl.ac.uk

Supplementary Information

S1. Synthesis of nanomaterials

S1.1. Materials

Gold(III) chloride trihydrate ($HAuCl_4 \cdot 3H_2O$ ≥99.9%), citric acid monohydrate (ACS reagent grade ≥99.0%), heptane (anhydrous, 99%), Tris-base (Trizma), hydrogen peroxide (H_2O_2, 30%wt), L-cysteine, silver nitrate ($AgNO_3$, 0.01 M) and NaOH (2M) were purchased from Sigma Aldrich. Trisodium citrate dihydrate (99%) was purchased from Alfa-Aesar. Hydroxylamine hydrochloride (≥99%) was purchased from Fisher Scientific. Carbon monoxide (CO) was obtained from BOC gases.

S1.2. Synthesis of Au and Au/Ag nanoclusters

Atomically precise $Au_{25}Cys_{18}$ nanoclusters were prepared via a flow chemistry approach, using a flow method developed in our group with some modifications.[1] An aqueous solution containing $HAuCl_4$ (final concentration 10 mM) and L-cysteine ($HAuCl_4$: L-cysteine, molar ratio 1:1.5) was prepared, and its pH was adjusted to 11.6 using NaOH. This solution was pumped through a tube-in-tube membrane reactor at a temperature of 80 °C, where CO diffused through the membrane and was dissolved in the solution. CO acts as reducing agent leading to the formation of the gold nanoclusters. The tube-in-tube membrane reactor consisted of a 2 m long Teflon AF-2400 tube (0.8 mm inner diameter (ID), 1.0 mm outer diameter (OD), Biogeneral) as the contacting interface placed inside a polytetrafluoroethylene (PTFE) tube (2.4 mm ID, 3.2 mm OD, VICI Jour) and assembled together by a T-piece (Upchurch, hole size: 1.0 mm) and a union (Upchurch, hole size: 1.0 mm). The solution of gold and cysteine was pumped using a milliGAT pump (VICI Valco) at a flow rate of 5 μL/s (average residence time inside the reactor of 3 min). The liquid inlet was pressurised to 5 bar using a Zaiput back pressure regulator (Zaiput) and monitored with a pressure sensor (Little Things Factory), while the gas inlet pressure was set at 4 bar with a gas pressure regulator (Swagelok, K series) and monitored with a pressure sensor (40PC150G, Honeywell). Gold/silver nanoclusters were prepared in the same system by only changing the liquid feed composition. For these clusters, a 23 mL aqueous solution containing $HAuCl_4$ and $AgNO_3$ and L-cysteine was prepared, then 2 mL of NaOH (2 M) was added to this solution. The final concentration of the sum of gold and silver precursors was equal to 10 mM, with a molar ratio of $HAuCl_4$: $AgNO_3$ equal to 16:9. The molar ratio between gold and silver precursors and L-cysteine $HAuCl_4$+$AgNO_3$: L-cysteine was 1:1.5. The colloidal solutions of gold and gold/silver clusters were stored in the fridge before use for no more than 2 days.

S1.3. Citrate-capped 11 nm gold nanoparticles

Citrate-capped 11 nm gold nanoparticles were synthesised via a flow chemistry approach according to Panariello et al..[2] A solution of passivated gold precursor was prepared the day before the experiment by adding NaOH to a solution of $HAuCl_4$ (gold concentration 8 mM, NaOH : $HAuCl_4$ molar ratio 27 : 1). This solution was then diluted to a final gold concentration of 0.4 mM with DI water and used as gold precursor. A solution of citric acid (4.8 mM) was used as reducing agent. The two aqueous solutions were pumped through a T-mixer (Upchurch, 0.5 mm internal diameter) at a volumetric ratio of 1:1, and then segmented with heptane in a second T-mixer (Upchurch, 0.5 mm internal diameter), generating a trail of moving droplets. The droplets then entered a heated coil (PTFE, 1 mm internal diameter, Thames Restek) at a temperature of 90 °C for an average residence time of 7 min, where the synthesis of the gold nanoparticles took place. The aqueous solution containing the 11 nm gold nanoparticles solution was separated by the heptane through decantation and pipetting once the reactor operation terminated. Subsequently, the colloidal solution was transferred to the fridge before further use.

S1.4. Tris-capped 50 nm gold nanoparticles

Tris-capped 50 nm gold nanoparticles were synthesised via a flow chemistry approach developed in our group,[3] where 11 nm gold nanoparticles (obtained as described in section S1.3) were used as seeds and grown up to 50 nm using the seeded-growth method reported by Li et al..[4] A solution of 11 nm gold seeds (colloidal Au(0) concentration of 0.06 mM and Tris (7.5 mM)) and a solution of $HAuCl_4$ (1.875 mM) were pumped through a T-mixer (Upchurch, 0.5 mm internal diameter), each one at a flow rate of 0.055 mL/min. This stream was then mixed with a H_2O_2 stream (1.85% wt) and segmented with a heptane stream within the same cross-junction (Upchurch, 0.5 mm internal diameter). The flow rate of H_2O_2 was set at 0.55 mL/min, while the flow rate of heptane was set at 0.165 mL/min. The droplets then entered an 11 mL PTFE coil (2.4 mm internal diameter) at room temperature, where the growth took place. A two-channel syringe pump (Legato® 200, KD Scientific) was employed to deliver the solutions of gold precursor and seeds, while two piston pumps (milliGAT, VICI Valco) were used for the H_2O_2 and the heptane solutions. The colloidal solution of 50 nm gold nanoparticles was manually separated from heptane through decantation and pipetting, once the reactor run terminated. Subsequently, the colloidal solution was transferred to the fridge before further use.

S1.5. Seeded-growth synthesis of citrate-capped 25 nm gold nanoparticles using citrate as reducing agent

Citrate-capped 25 nm gold nanoparticles were prepared through a seeded-growth approach using 11 nm gold nanoparticles as seeds (synthesised as in section S1.3). 18 mL of an aqueous solution of 11 nm gold seeds (colloidal Au(0) concentration 0.11 mM) was preheated to 90 °C in a stirred three-neck round-bottom flask. To this solution, 1 mL of an 8 mM solution of passivated gold precursor (prepared as in section S1.3) was added. After 5 min, 1 mL of a 72 mM citric acid solution was injected in the flask. After 10 min, the flask was removed from the hot bath and allowed to cool to room temperature. Subsequently, the colloidal solution was transferred to the fridge before further use.

S1.6. Seeded-growth synthesis of citrate-capped 20 and 50 nm gold nanoparticles using hydroxylamine as reducing agent

Citrate-capped 20 and 50 nm gold nanoparticles were prepared through a seeded growth protocol proposed by Brown and Natan[5] using hydroxylamine as reducing agent. 11 nm gold nanoparticles were used as seeds, synthesised as in section S1.3. For the synthesis of 20 nm particles, 2 mL of an aqueous solution of 11 nm gold seeds (colloidal Au(0) concentration 0.2 mM) was diluted with 1.895 mL of DI

water. 0.057 mL of a 0.2 M hydroxylamine hydrochloride solution was then added to the solution under vigorous stirring. After 1 min, 0.048 mL of a 25 mM $HAuCl_4$ solution was added under vigorous stirring at room temperature. The solution immediately turned from light to dark red. Stirring was maintained for 5 min, then the colloidal solution was transferred to the fridge before further use. For the synthesis of 50 nm particles, an iterative procedure was adopted. A first growth step was performed, where 1.25 mL of an aqueous solution of 11 nm gold seeds (colloidal Au(0) concentration 0.2 mM) was diluted with 23.4 mL of DI water. 0.35 mL of 0.2 M hydroxylamine hydrochloride solution was then added to the solution under vigorous stirring. After 1 min, 0.3 mL of a 25 mM $HAuCl_4$ solution was added at room temperature. Stirring was maintained for 5 min. After the first growth step, 9.867 mL of the colloidal solution synthesised was diluted with 3.08 mL of DI water, and to this solution 0.188 mL of a 0.2 M hydroxylamine hydrochloride solution was added under stirring. After 1 min, 0.16 mL of a 25 mM $HAuCl_4$ solution was injected. Stirring was maintained for 5 min, then the colloidal solution was transferred to the fridge before further use.

S2. Dye molecule surface area calculation

To determine the van der Waals surface area of dye molecules, Multiwfn software[6] was employed. This model was based on promolecular density. Solvent accessible surface area and space filling projection surface areas were calculated using Visual Molecular Dynamic (VMD) software.[7] The formulae for calculating the dye concentration required for monolayer coverage of the AuNP surface is: [8]

$$N(\mathrm{mL}^{-1}) = \frac{A_{450}\times 10^{14}}{d^2\left[-0.295+1.36\,exp\left(-\left(\frac{d-96.8}{78.2}\right)^2\right)\right]} \quad (1)$$

$$[\mathrm{dye}](\mu\mathrm{M}) = \frac{N\pi d^2\times 10^9}{SN_A} \quad (2)$$

N is the number density of AuNP solutions, A_{450} is the absorbance at λ = 450 nm and *d* is the particle diameter in nm. *S* is the surface area of dye projected onto the AuNP. For VDWSA and SASA methods, *S* is half of the dye surface area. N_A is the Avogadro constant.

S3. Nanoparticle number density

In addition to using Eq. (1) to determine the number density of nanoparticles, we also tested the method introduced by Hendel et. al.[9] Their method uses the extinction coefficient ε at 400 nm for 20 nm AuNPs synthesized by reduction with sodium citrate which is 2.38 L $mmol^{-1}$ cm^{-1},

$$N(\mathrm{mL}^{-1}) = \frac{A_{400}M_{Au}\times 10^{15}}{\frac{\pi d^3}{6}\varepsilon b\rho_{Au}} \quad (3)$$

Where *N* is the number density of AuNP solutions, A_{400} is the absorbance at λ = 400 nm, M_{Au} is the molar mass of gold, *b* is the light path length, *d* is the particle diameter in nm, ε is the extinction coefficient in L $mmol^{-1}$ cm^{-1} and ρ_{Au} is the density of gold. From Fig. 1 in the main manuscript, we deduce that A_{400} = 0.51 and 0.49 for CV and MB, respectively, giving $N = 4.66 \times 10^{11}$ mL^{-1} and 4.50×10^{11} mL^{-1} for CV and MB, respectively. The surface area of CV is estimated to be 7.46 nm^2 (VDWSA), 10.56 nm^2 (SASA) or 1.72 nm^2 (SFP), and that of MB is estimated to be, 3.63 nm^2 (VDWSA), 6.01 nm^2 (SASA) or 0.93 nm^2 (SFP). From these values, we can calculate the concentrations of dye required for monolayer coverage of the AuNPs and compare these with the threshold concentrations determined from the UV-vis spectra (Table S1); we find that they are very similar to those obtained using Haiss's method.

Table S1. Comparison between the calculated dye concentration required (using Hendel's model) for monolayer coverage of the AuNP surface compared with the threshold concentration determined from UV-vis absorption spectra (Fig. 1 in the main manuscript).

		20 nm citrate-capped AuNP	
	Model	MB	CV
Concentration of dye / µM	VDWSA	0.52	0.26
	SASA	0.31	0.19
	SFP	1.01	0.57
Experimental threshold conc. / µM		0.55	0.36
Corresponding solution Vol. / mL		6.0	5.0

S4. Additional data

Table S2. Particle size measurement for TEM images. Analysed by imageJ, only counting monomers and not including agglomerated particles.

MB (mL)	Size (nm)	Std. dev (nm)	Particle count	CV (mL)	Size (nm)	Std. dev (nm)	Particle count
0	19.60	3.68	48	0	19.60	3.68	48
5	20.94	5.06	33	5	21.34	7.30	131
10	22.08	6.18	73	10	19.30	3.59	51
15	Not enough monomer			15	Not enough monomer		

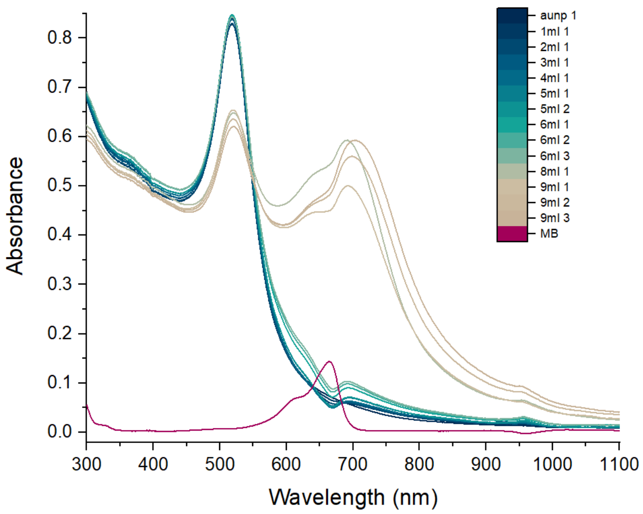

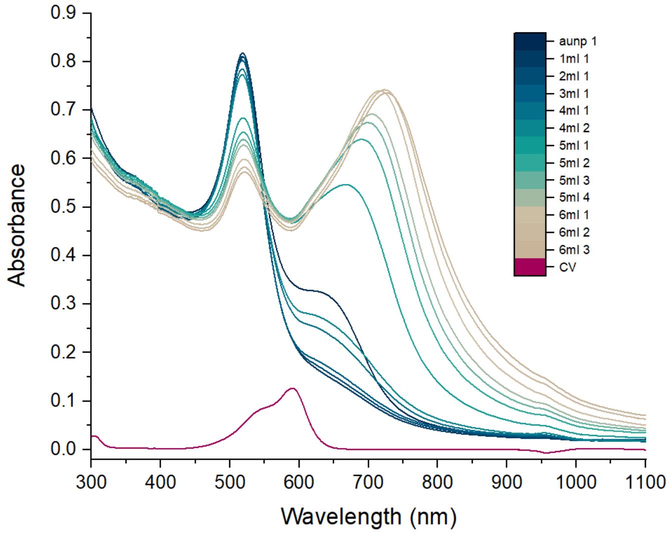

Figure S1. UV-vis absorption of 2 mM MB (top) and 1.5 mM MB (bottom) in solutions of 20 nm citrate-capped AuNPs (Figure 1). The red curve is the absorption spectrum of the dye. The other curves represent spectra recorded following the addition of 1 - 9 ml dye solution (the numbers at the ends of the labels in the key state the measurement number) with the dye contribution subtracted and spectra corrected for dilution effects.

References

1. A. Gavriilidis, I. P. Parkin, G. B. Hwang, G. Wu, J. Shin, L. Panariello, V. Sebastian, K. Karu and E. Allan, *ACS Appl. Mater. Interfaces*, 2020, **12**, 49021–49029.
2. L. Panariello, S. Damilos, H. Du Toit, G. Wu, A. N. P. Radhakrishnan, I. P. Parkin and A. Gavriilidis, *React. Chem. Eng.*, 2020, **5**, 663–676.
3. L. Panariello, K. Chuen To, Z. Khan, G. Wu, G. Gkogkos, S. Damilos, I. P. Parkin and A. Gavriilidis, *Chem. Eng. J.*, 2021, 129069.
4. Y. Li, P. Zhang, J. Duan, S. Ai and H. Li, *CrystEngComm*, 2017, **19**, 318–324.
5. K. R. Brown and M. J. Natan, *Langmuir*, 1998, **14**, 726–728.
6. T. Lu and F. Chen, *J. Comput. Chem.*, 2012, **33**, 580–592.
7. W. Humphrey, A. Dalke and K. Schulten, *J. Mol. Graph.*, 1996, **14**, 33–38.
8. W. Haiss, N. T. K. Thanh, J. Aveyard and D. G. Fernig, *Anal. Chem.*, 2007, **79**, 4215–4221.
9. T. Hendel, M. Wuithschick, F. Kettemann, A. Birnbaum, K. Rademann and J. Polte, *Anal. Chem.*, 2014, **86**, 11115–11124.
